# A Draft Genome Assembly of *Culex pipiens pallens* (Diptera: Culicidae) Using PacBio Sequencing

**DOI:** 10.1093/gbe/evab005

**Published:** 2021-01-27

**Authors:** Cheng Peng, Zhang Qian, Zhang Xinyu, Le Qianqian, Gong Maoqing, Zhang Zhong, Zhang Ruiling

**Affiliations:** 1 Collaborative Innovation Center for the Origin and Control of Emerging Infectious Diseases, Shandong First Medical University (Shandong Academy of Medical Sciences), Tai’an, China; 2 Shandong Institute of Parasitic Diseases, Shandong First Medical University (Shandong Academy of Medical Sciences), Jining, China; 3 School of Basic Medical Sciences, Shandong First Medical University (Shandong Academy of Medical Sciences), Tai’an, China

**Keywords:** Culicidae, genome annotation, comparative genomics, gene family evolution, vector

## Abstract

The Northern house mosquito, *Culex pipiens pallens*, serves as important temperate vectors of several diseases, particularly the epidemic encephalitis and lymphatic filariasis. Reference genome of the *Cx. pipiens pallens* is helpful to understand its genomic basis underlying the complexity of mosquito biology. Using 142 Gb (∼250×) of the PacBio long reads, we assembled a draft genome of 567.56 Mb. The assembly includes 1,714 contigs with a N50 length of 0.84 Mb and a Benchmarking Universal Single-Copy Orthologs (BUSCO) completeness of 95.6% (*n* = 1,367). We masked 60.63% (344.11 Mb) of the genome as repetitive elements and identified 2,032 noncoding RNAs. A total of 18,122 protein-coding genes captured a 94.1% of BUSCO gene set. Gene family evolution and function enrichment analyses revealed that significantly expanded gene families mainly involved in immunity, gustatory and olfactory chemosensation, and DNA replication/repair.

**Significance**

Mosquitoes are important vectors of many pathogens and causing heavy threats to public health and economy worldwide. Whole-genome sequencing of 30 mosquito species (27 *Anopheles*, 2 *Aedes*) has been reported, whereas there was only one *Culex* genome available presently. In this study, we generated the draft genome of *Culex pipiens pallens*, which is the primary vector of lymphatic filariasis, epidemic encephalitis and widely distributed in northern China. The genome assembly of *Cx. pipiens pallens* was 567.56 Mb. Reference genome of *Cx. pipiens pallens* would help to understanding its genomic basis underlying the complexity of mosquito biology.

## Introduction

Mosquitoes are important vectors that can transmit a variety of infectious diseases and more than half of the world’s population is threatened by mosquito-borne diseases, causing a huge burden to human health and the economy (Gething et al. 2011, 2012; Bhatt et al. 2013; Acevedo et al. 2015). Species of *Culex pipiens* complex (Diptera: Culicidae) are globally distributed and has been considered as major vectors of several diseases. Members of this complex including *Culex quinquefasciatus*, *Cx. pipiens pallens*, *Culex pipiens pipiens*, *Culex pipiens molestus*, *Culex australicus*, and *Culex globocoxitus* (Smith and Fonseca 2004; Harbach 2012; Russell 2012; Aardema et al. 2020). *Culex pipiens pallens* is the most widely distributed subspecies of *Cx. pipiens* complex in northern China and the primary vector of lymphatic filariasis, epidemic encephalitis (Fonseca et al. 2009; Turell 2012; Cano et al. 2014).

High-quality mosquito genomes are the important genetic resources for the studies of vector-borne biology and evolutionary of bloodsucking characters. To date, 30 mosquitoes (Culicidae) genomes have been public (NCBI, accessed November 25, 2020), including 27 *Anopheles*, 2 *Aedes*, and 1 *Culex* species. The assembly sizes (ca. 150–300 Mb) of *Anopheles* genomes are rather smaller than other two genera (>500 Mb in *Culex* and >1 Gb in *Aedes*). The only available *Cx. quinquefasciatus* genome has an assembly size of 579.04 (539.96 ungapped) Mb, 48,671 contigs and 3,171 scaffolds, and a contig/scaffold N50 length of 28.55/486.76 kb (table 1, Arensburger et al. 2010). Here, we assembled a de novo genome assembly of *Cx. pipiens pallens* using the Pacific Bioscience (PacBio) single-molecule real-time (SMRT) platform. We annotated the protein-coding genes, as well as repetitive elements and non-coding RNAs (ncRNAs). Gene family evolution across the main Diptera clades was analyzed, particularly focusing on those rapidly evolving families.

**Table 1:** Genome Assembly and Annotation Statistics of Two *Culex* Species

	*Culex pipiens pallens*	*Culex quinquefasciatus*
Genome assembly		
Assembly size (Mb)	567.56	579.04
Number of scaffolds/contigs	1,714/1,714	3,171/48,671
Longest scaffold/contig (Mb)	6.08/6.08	3.87/0.43
N50 scaffold/contig length (kb)	839.22/839.22	486.76/0.0286
GC (%)	36.76	37.42
Gaps (%)	0.00	6.75%
BUSCO completeness (%)	95.61	95.68
Gene annotation		
Protein-coding genes	18,122	18,883
Mean protein length (aa)	500.59	436.43
Mean gene length (bp)	6,695.83	5,687.53
Exons per gene	3.68	3.96
Exon (%)	5.22	4.38
Mean exon length	444.61	356.74
Intron (%)	16.15	14.17
Mean intron length	1,972.73	1,579.51
BUSCO completeness (%)	94.07	94.59

## Materials and Methods

### Sample Collection and Sequencing

The *Cx. pipiens pallens* strain used for sequencing was originally collected from Mengtougou (China, Beijing) in 1999, and has been maintained in the laboratory without exposure to any insecticides. Female adults emerging from pupae without feeding were prepared for sequencing: 100 for Illumina and PacBio whole genome and 50 for transcriptome, respectively. We extracted genomic DNA using the Qiagen Blood and CELL Culture DNA mini Kit, constructed a library of 350 bp insert size using the TruSeq DNA PCR-Free LT Library Preparation Kit and a library of a 40 kb-insert size using a SMRTbell DNA Template Prep Kit 2.0. Genomic RNA was extracted using TRIzol Reagent and library was constructed using the TruSeq RNA v2 Kit. Short-read libraries were subject to the paired-end 150 bp (PE 150) sequencing on the HiSeq NovaSeq 6000 platform. Long-read library was sequenced on the PacBio Sequel II system using the Sequel Sequencing kit v2.1 chemistry. All libraries were sequenced at Berry Genomics (Beijing, China).

### Genome Assembly

Quality control of Illumina sequences including removal of duplicates using “clumpify.sh,” adapter trimming, quality trimming (>Q20), polymer trimming (>10 bp for poly-A/G/C tails), length filtering (>15 bp), and correction of overlapping paired reads using “bbduk.sh” were performed using BBTools suite v38.67 (Bushnell 2014).

Preliminary genome assembly and long-read polishing were performed using Flye v2.7 (Kolmogorov et al. 2019) with a minimum overlap between reads of 5,000, 50× longest reads for an initial contig assembly and one round of self-polishing (‘-m 5000 --asm-coverage 50 -i 1’). Redundant heterozygous contigs were removed using three rounds of Purge_Dups v1.0.0 (Guan et al. 2020) based on read depth with a minimum alignment score of 50 and a minimum chaining score of 5,000 for a match (‘-a 50 -l 5000’). Resulting non-redundant assembly was polished with Illumina short reads using two rounds of NextPolish v1.1.0 (Hu et al. 2020). Minimap2 v2.12 (Li 2018) was used as sequence aligner for above redundancy removal and short-read polishing. We detected potential contaminant sequences using HS-BLASTN (high-speed blastn, Chen et al. 2015) against the NCBI nucleotide (nt) and UniVec databases. To assess the assembly quality, we assessed genome completeness using Benchmarking Universal Single-Copy Orthologs (BUSCO) v3.0.2 pipeline (Waterhouse et al. 2018) against insect reference gene set (*n* = 1,367), and calculated the mapping rate by aligning PacBio long reads and Illumina short reads to the final genome assembly with Minimap2.

### Genome Annotation

Three essential elements, including repetitive elements, ncRNAs and protein-coding genes were annotated for the genome of *Cx. pipiens pallens*. To annotate repeats in the genome, we constructed a de novo repeat library using RepeatModeler v2.0.1 (Flynn et al. 2020) with new LTR discovery pipeline included (“-LTRStruct”), and then combined it with Dfam_3.1(Hubley et al. 2016) and RepBase-20181026 databases (Bao et al. 2015) to generate a custom library. Repetitive elements were masked in the genome using RepeatMasker v4.0.9 (Smit et al. 2013–2015) based on above custom library. ncRNAs were identified using Infernal v1.1.2 (Nawrocki and Eddy 2013); tRNAs were refined using tRNAscan-SE v2.0.6 (Chan and Lowe 2019) with only those of high confidence kept by tRNAscan-SE script “EukHighConfidenceFilter.”

3MAKER v3.01.03 pipeline (Holt and Yandell 2011) was used to predict protein-coding genes by integrating ab initio, transcript- and protein homology-based evidence. The current version of MAKER also used the idea of evidence weights from EVidenceModeler (EVM). Ab initio predictions were constructed using BRAKER v2.1.5 pipeline (Hoff et al. 2016), which generated gene structure annotations by automatically training the predictors Augustus v3.3.2 (Stanke et al. 2004) and GeneMark-ES/ET/EP 4.48_3.60_lic (Lomsadze et al. 2005) incorporating evidence from transcriptome and protein homology information. RNA-seq information were provided as BAM alignments produced using HISAT2 v2.2.0 (Kim et al. 2019); arthropod protein sequences were extracted from OrthoDB10 v1 database and passed to BRAKER (Kriventseva et al. 2019). We assembled transcripts using genome-guided assembler StringTie v2.1.4 (Kovaka et al. 2019). Protein sequences of *Drosophila melanogaster*, *Cx. quinquefasciatus*, *Aedes aegypti*, *Anopheles gambiae*, and *Tribolium castaneum* were downloaded from the NCBI as the protein homology evidence for MAKER annotation. Evidence weights were set as 1, 2, 10 for ab initio, protein and transcript evidence, respectively. Gene functions were annotated by searching UniProtKB database using Diamond v0.9.24 (Buchfink et al. 2015) with the sensitive mode “--more-sensitive -e 1e-5.” We annotated protein domains, Gene Ontology (GO) and pathways (KEGG, Reactome) using InterProScan 5.41-78.0 (Finn et al. 2017) against Pfam (EI-Gebali et al. 2019), Panther (Mi et al. 2019), Gene3D (Lewis et al. 2018), Superfamily (Wilson et al. 2009), SMART (Letunic and Bork 2018), and CDD (Marchler-Bauer et al. 2017) databases, and using eggNOG-mapper v2.0.1 (Huerta-Cepas et al. 2017) against the eggNOG v5.0 database (Huerta-Cepas et al. 2019).

### Phylogenomics and Gene Family Evolution

Twelve Diptera species (Culicoidea: *Ae. aegypti*, *Aedes albopictus*, *An. gambiae*, *Anopheles stephensi*, *Cx. pipiens pallens*, *Cx. quinquefasciatus*; Chironomoidea: *Belgica antarctica*, *Culicoides sonorensis*; Sciaroidea: *Contarinia nasturtii*; Ephydroidea: *D. melanogaster*; Oestroidea: *Lucilia cuprina*; Muscoidea: *Musca domestica*), one Coleoptera species (*T. castaneum*), one Lepidoptera species (*Bombyx mori*) were selected for orthology inference using OrthoFinder v2.3.8 (Emms and Kelly 2019) with Diamond as the sequence aligner. Protein sequences of *B. antarctica* and *C. sonorensis* were downloaded from Ensembl with others from the NCBI.

Resulting single-copy orthologs were used for phylogenetic inference. Protein sequences were aligned using MAFFT v7.394 (Katoh and Standley 2013) with the L-INS-I strategy, trimmed unreliable homologous regions using BMGE v1.12 (Criscuolo and Gribaldo 2010) with the stringent parameters “-m BLOSUM90 -h 0.4,” concatenated loci alignments using FASconCAT-G v1.04 (Kück and Longo 2014), and inferred a phylogenetic tree using IQ-TREE v2.0-rc1 (Minh et al. 2020) with the partitioning strategy (“-m MFP --mset LG --msub nuclear --rclusterf 10 -B 1000 --alrt 1000 --symtest-remove-bad --symtest-pval 0.10”). We estimated divergence time using MCMCTree, a tool within the PAML v4.9j package (Yang 2007). Four fossil node calibration information were obtained from the PBDB database (https://www.paleobiodb.org/navigator/): root (<350 Ma), Holometabola (311.4–3.232 Ma), Chironomidae (201.3–208.5 Ma), and Culicidae (93.5–100.5 Ma).

We estimated expansions and contractions of gene families using CAFÉ v4.2.1 (Han et al. 2013) with the approach of single birth–death parameter lambda and the significance level of 0.01. Function enrichment analyses of GO and KEGG categories were also performed for those significantly expanded families using R package clusterProfiler v3.10.1 (Yu et al. 2012) with the default significance values (*P*-value <0.01 and *q*-value <0.05).

## Results and Discussion

### Genome Assembly

Altogether, 140.47 Gb (∼246×) Illumina short reads and 142.74 Gb (∼250×) PacBio subreads and 6.59 Gb transcriptome data were generated. The mean and N50 length of the long PacBio subreads are 22,438.45 kb and 34.59 kb, respectively. After quality control, 124.07 Gb (∼218×) short reads were kept for the subsequent genome polishing.

Raw PacBio reads were assembled into 10,627 contigs by Flye and the obtained 1.7 Gb length of sequences almost reached three times size of *Cx. quinquefasciatus* genome. Although completed BUSCOs occupied 99.1%, the BUSCO completeness assessment (*n* = 1,367) identified 1,270 (92.9%) reference genes as complete and duplicated BUSCOs. This result indicated that Flye assembly possessed a very high ratio of redundancy. After redundancy removal, polishing and contaminant detection, the final *Cx. pipiens pallens* assembly had a length of 567.56 Mb, which comprising 1,714 contigs. The contig N50 length was 839.22 kb, GC content was 36.76%, and BUSCO completeness was 95.6% (5% complete and duplicated, 0.4% fragmented, 4% missing). The assembly size and low ratio of redundancy (5%) suggested that most heterogeneous contigs had been successfully removed from the assembly of *Cx. pipiens pallens*. Overall, the assembly size of *Cx. pipiens pallens* was closer to that of *Cx. quinquefasciatus*, as well as an estimate of 540 Mb from reassociation kinetics (Rao and Rai 2008). However, compared with the *Cx. pipiens pallens* assembly in this study, the genome of congeneric *Cx. quinquefasciatus* had much lower contig contiguity and a high level of gaps (table 1). Genome alignment at a 0.1% sequence divergence (-asm5) using Minimap2 showed that 99.99% CPP assembly regions could be mapped to the genome of *Cx. quinquefasciatus*.

### Genome Annotation

More than half (60.63%, 344.11 Mb) of the genome were masked as repetitive elements. The top five abundant repeat categories were DNA elements (29.68%), unclassified (13.49%), LTR (6.19%), LINE (4.39%), and simple repeats (3.89%) (supplementary table S1, Supplementary Material online). Among DNA transposon groups, *Sola-2* (9.02%) and *Zator* (3.13%) were the two largest superfamilies encoding DDD- and DDE-transposases, respectively (Bao et al. 2009). A large amount of repeat content may be the important resource of *Culex* genome expansion compared with *Anopheles*.

Altogether, 2,032 ncRNAs were identified using Infernal and tRNAscan, including 185 rRNAs, 68 miRNAs, 67 small nuclear RNAs (snRNAs), 2 long non-coding RNAs (lncRNAs), 647 tRNAs (22 isotypes), 27 ribzymes, and 1,035 other ncRNAs (supplementary table S2, Supplementary Material online). snRNAs were classified as 53 spliceosomal RNAs (U1, U2, U4, U5, U6, U11), three minor spliceosomal RNAs (U4atac, U6atac, U12), eight C/D box snoRNAs (U3, snoMe28S-Am2589, snosnR60_Z15, snoU18), and one H/ACA box snoRNA (snoR639). A large number (1,018) of histone 3′ UTR stem-loop RNAs were also discovered in the *Cx. pipiens pallens* genome.

We predicted 18,122 protein-coding gene models using MAKER pipeline. Compare with *Cx. quinquefasciatus*, gene predictions of *Cx. pipiens pallens* had longer mean lengths of genes, exons and introns (table 1), indicating that high-quality gene prediction in this study. BUSCO completeness assessment identified 94.1% complete genes (*n* = 1,367) using protein mode “-m prot.” Diamond searches aligned 17,104 (94.38%) genes to the UniprotKB records. Protein domain and function annotations assigned protein domains of 14,564 (80.37%) genes, 12,788 GO terms, 8,484 KEGG ko terms, 2,740 Enzyme Codes, 5,014 KEGG and 3,548 Reactome pathways, and 14,867 COG categories, respectively.

### Phylogeny

**fig. 1.**
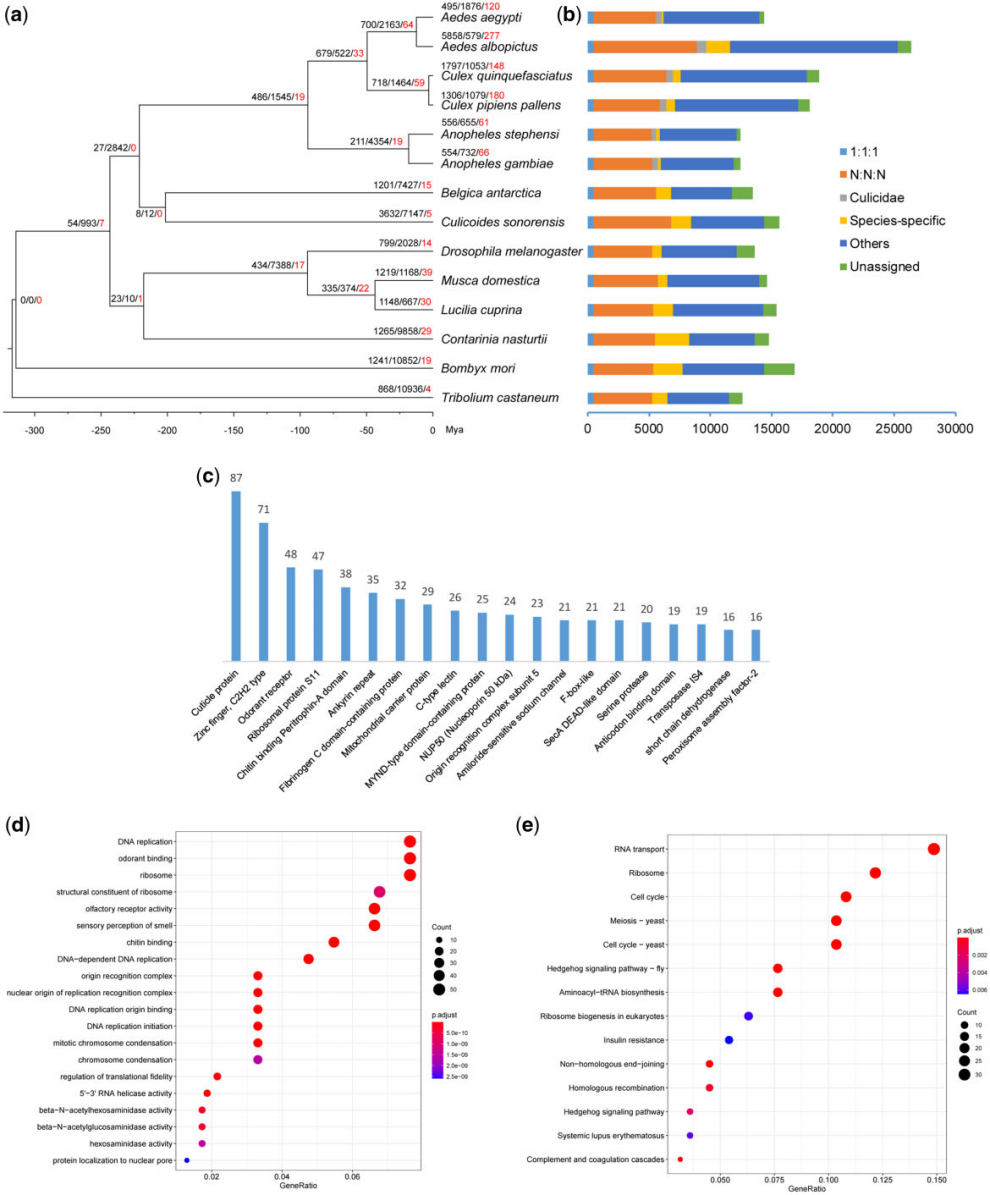
—Phylogeny, orthologs, and gene family evolution. (*a*) Phylogeny, dating and gene family evolution. Node values representing the number of expanded, contracted and rapidly evolving families, respectively. (*b*) Statistics of orthologs and paralogs. “1:1:1” represents shared single-copy genes, “N:N:N” as multi-copy genes shared by all species, “Culicidae” as orthologs unique to Culicidae, “Others” as unclassified orthologs, “Unassigned” as orthologs which cannot be assigned into any orthogroups. (*c*) Top twenty significantly expanded families with gene numbers of the families shown above the bars. (*d*) and (*e*) Function enrichment of GO (*d*) and KEGG (*e*) for significantly expanded gene families. Only the top twenty categories are shown.

OrthoFinder clustered 204,879 (93.16%) genes into 18,992 gene families (orthogroups). Among 4,150 orthogroups with all species present, 440 are single-copy ones. 344 families and 3,243 orthologs are unique to six Culicidae species (fig. 1*b*, supplementary table S3, Supplementary Material online). For *Cx. pipiens pallens*, 17,195 (94.88%) genes were clustered into 11,352 orthogroups; among them, 208 orthogroups and 696 genes were species-specific.

Thirty-six single-copy genes were removed by “symtest” in IQ-TREE and remaining 404 genes were used to phylogenetic inference. Phylogenetic reconstruction based on 155,458 amino acid sites was fully resolved with 100/100 node supports. Topology, that is, classification was also consistent with previous studies (Wiegmann et al. 2011). Culicomorpha (Culicoidea + Chironomoidea) was sister to other dipteran species. Dating analyses revealed that stem of Culicidae originated from late Triassic (218.51–224.15 Ma). It almost emerged simultaneously with mammals and dinosaurs, which may provide opportunities of evolution of new feeding habits (i.e., sucking blood) for mosquitoes. Two *Culex* species diverged from late Neogene (2.72–3.54 Ma) (fig. 1*a*).

### Gene Family Evolution

Gene family evolution estimated using CAFÉ upon phylogenetic tree is shown in figure 1*a*. For *Cx. pipiens pallens*, there were 1,306 and 1,079 gene families experienced expansions and contractions, respectively. Among all of the changed genes, 180 genes (125 expansions and 55 contractions) were rapidly evolving gene families. Most significant expanded families were related to immunity, gustatory and olfactory chemosensation, and DNA replication/repair. Immunity-related families included chitin binding Peritrophin-A domain-containing protein (Terra 2001), Fibrinogen C domain-containing protein (von Huth et al. 2018), C-type lectin (Brown et al. 2018), helicase MOV (Balinsky et al. 2017), and Peptidoglycan recognition protein (Royet et al. 2011). Origin recognition complex subunit 5 (ORC5), mitochondrial DNA polymerase (containing anticodon binding domain) and Geminin genes involve in DNA replication and repair (Lee et al. 2009; Copeland 2010; Tang et al. 2017; Coulombe et al. 2019). Large expansions of immune- and DNA replication/repair-related genes explained the possible mechanism of adaptations to polluted, harsh environment for mosquitoes, particularly their larvae. In *Cx. quinquefasciatus*, Arensburger et al. (2010) discovered the expansions of cytosolic glutathione transferases and cytochrome P450s adaptable to evasion of insecticides, but not the case in *Cx. pipiens pallens*. Mosquito chemosensation are crucial for host seeking, foraging, mating, and oviposition (Clements 1992), the expansions of gustatory and olfactory receptors may reflect olfactory behavioral diversity of the *Cx. pipiens pallens* in host and oviposition site choice (Kwon et al. 2006; Sparks et al. 2018). Further enrichment analyses of GO (fig. 1*d*) and KEGG (fig. 1*e*) for those significant expanded gene families also reinforced above results, such as GO categories: chemosensation-related (odorant binding, olfactory receptor activity, sensory perception of smell), DNA replication-related (DNA replication, DNA-dependent DNA replication, DNA replication origin binding, DNA replication initiation, chromosome condensation).

This is the first genome assembly for *Cx. pipiens pallens*. Considering the importance of *Cx. pipiens pallens* as a vector of several human pathogens, we hope insights from the genome resource will be helpful for advance the understanding of biological characters of this species and contribute to ongoing efforts to develop control measures of mosquitoes and mosquito-borne diseases.

## Supplementary Material

Supplementary data are available at *Genome Biology and Evolution* online.

## Supplementary Material

evab005_Supplementary_Data
