# Supplementary material for: A Draft Genome Assembly of Culex pipiens pallens (Diptera: Culicidae) Using PacBio Sequencing
Source: Genome Biol Evol. 2021 Jan 27;13(3):evab005. doi: 10.1093/gbe/evab005 (PMC7936019; doi:10.1093/gbe/evab005)
Supplement: evab005_Supplementary_Data [file evab005_supplementary_data.zip › FigS1.pdf]

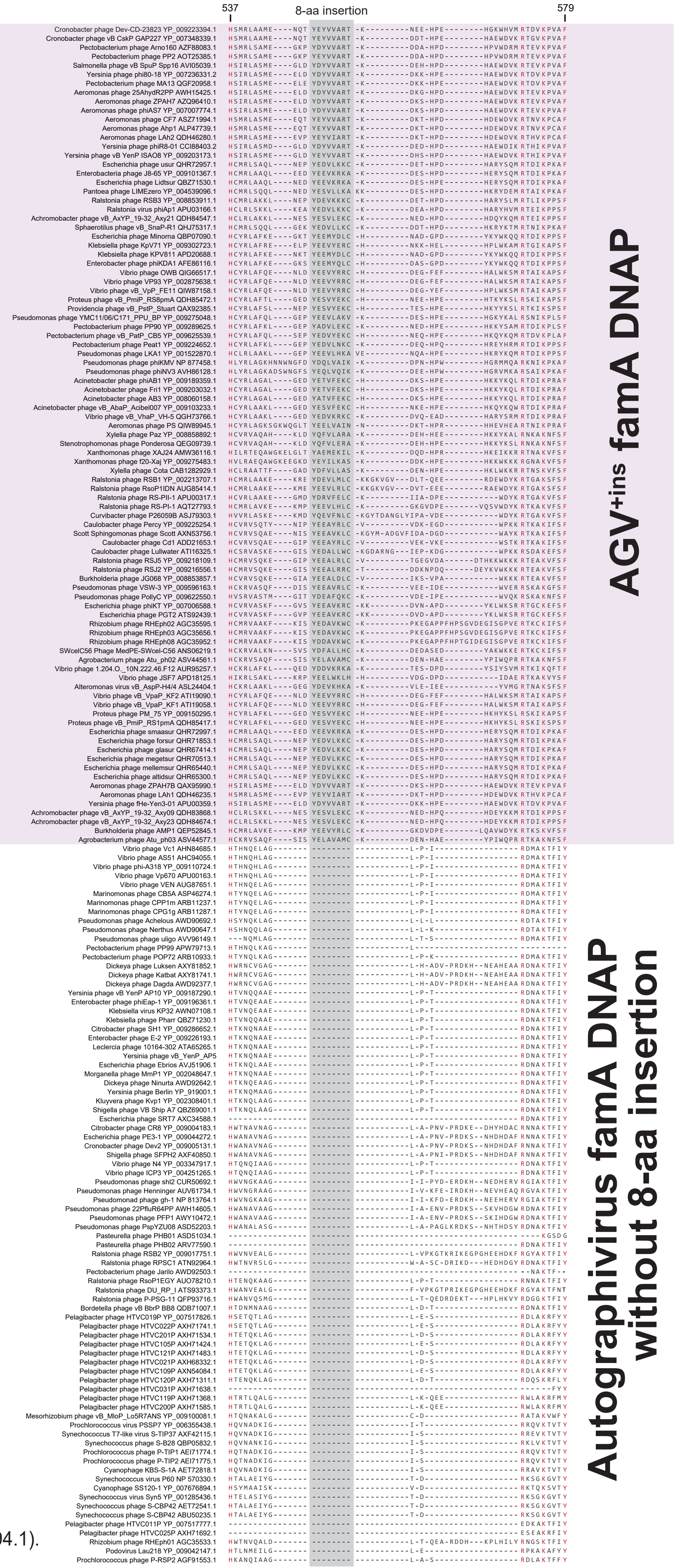

Fig. S1

Partial amino acid alignment of the portions containing 8-aa insertions in autographivirus famA DNAPs. 8-aa insertions are shaded in gray. The amino acid residue numbers shown on the left and right edges of the alignment are based on the famA DNAP of Cronobacter phage DevCD23823 (YP_009223394.1).
