# Supplementary material for: A Draft Genome Assembly of Culex pipiens pallens (Diptera: Culicidae) Using PacBio Sequencing
Source: Genome Biol Evol. 2021 Jan 27;13(3):evab005. doi: 10.1093/gbe/evab005 (PMC7936019; doi:10.1093/gbe/evab005)
Supplement: evab005_Supplementary_Data [file evab005_supplementary_data.zip › Table_S1.pdf]

subfamily	genus	species	GenBank accession no.	Presence/absence of 8-aa insertion	Phylogenetic affinity to PolIBCD+
Beijerinckvirinae	Friunavirus	Acinetobacter virus AB3	YP_008060158.1	yes	yes
Beijerinckvirinae	Friunavirus	Acinetobacter phage phiAB1	YP_009189359.1	yes	yes
Beijerinckvirinae	Friunavirus	Acinetobacter phage Fri1	YP_009203032.1	yes	yes
Colwellvirinae	Gutovirus	Vibrio phage Vc1	AHN84685.1	no	no
Colwellvirinae	Kaohsiungvirus	Vibrio phage AS51	AHC94055.1	no	no
Colwellvirinae	Kaohsiungvirus	Vibrio phage Vp670	APU00163.1	no	no
Colwellvirinae	Murciavirus	Marinomonas phage CB5A	ASP46274.1	no	no
Colwellvirinae	Murciavirus	Marinomonas phage CPP1m	ARB11237.1	no	no
Colwellvirinae	Trungvirus	Vibrio phage VEN	AUG87651.1	no	no
Colwellvirinae	Uliginvirus	Pseudomonas phage Achelous	AWD90692.1	no	no
Colwellvirinae	Uliginvirus	Pseudomonas phage Nerthus	AWD90647.1	no	no
Colwellvirinae	Uliginvirus	Pseudomonas phage uligo	AVV96149.1	no	no
Corkvirinae	Phimunavirus	Pectobacterium phage Peat1	YP_009224652.1	yes	yes
Corkvirinae	Phimunavirus	Pectobacterium phage vB_PatP_CB5	YP_009625539.1	yes	yes
Corkvirinae	Phimunavirus	Pectobacterium phage PP90	YP_009289625.1	yes	yes
Krylovirinae	Phikmvvirus	Pseudomonas phage LKA1	YP_001522870.1	yes	yes
Krylovirinae	Phikmvvirus	Pseudomonas phage phiKMV	NP_877458.1	yes	yes
Krylovirinae	Phikmvvirus	Pantoea phage LIMEzero	YP_004539096.1	yes	yes
Melnykvirinae	Aerosvirus	Aeromonas phage 25AhydR2PP	AWH15425.1	yes	yes
Melnykvirinae	Aerosvirus	Aeromonas phage ZPAH7	AZQ96410.1	yes	yes
Melnykvirinae	Ahphunavirus	Aeromonas phage Ahp1	ALP47739.1	yes	yes
Melnykvirinae	Ahphunavirus	Aeromonas phage CF7	ASZ71994.1	yes	yes
Melnykvirinae	Wanjuvirus	Pectobacterium phage Arno160	AZF88083.1	yes	yes
Melnykvirinae	Wanjuvirus	Pectobacterium phage PP2	AOT25385.1	yes	yes
Molineuxvirinae	Zindervirus	Pectobacterium phage POP72	ARB10933.1	no	no
Molineuxvirinae	Zindervirus	Marinomonas phage CPG1g	ARB11287.1	no	no
Molineuxvirinae	Zindervirus	Vibrio phage phi-A318	YP_009110724.1	no	no
Okabevirinae	Higashivirus	Ralstonia phage RSB1	YP_002213707.1	yes	yes
Okabevirinae	Higashivirus	Ralstonia phage RsoP1IDN	AUG85414.1	yes	yes
Okabevirinae	Mguuvirus	Burkholderia phage JG068	YP_008853857.1	yes	yes
Okabevirinae	Risjevirus	Ralstonia phage RSJ2	YP_009216556.1	yes	yes
Okabevirinae	Risjevirus	Ralstonia phage RSJ5	YP_009218109.1	yes	yes
Slopekvirinae	Drulisvirus	Escherichia phage Minorna	QBP07090.1	yes	yes
Slopekvirinae	Drulisvirus	Klebsiella phage KpV71	YP_009302723.1	yes	yes
Slopekvirinae	Drulisvirus	Klebsiella phage KPV811	APD20688.1	yes	yes
Studiervirinae	Aarhusvirus	Dickeya phage Luksen	AXY81852.1	no	no
Studiervirinae	Aarhusvirus	Dickeya phage Dagda	AWD92377.1	no	no
Studiervirinae	Aarhusvirus	Dickeya phage Katbat	AXY81741.1	no	no
Studiervirinae	Apdecimavirus	Yersinia phage vB_YenP_AP10	YP_009187290.1	no	no
Studiervirinae	Berlinvirus	Yersinia phage Berlin	YP_919001.1	no	no
Studiervirinae	Berlinvirus	Kluyvera phage Kvp1	YP_002308401.1	no	no
Studiervirinae	Berlinvirus	Shigella phage VB_Ship_A7	QBZ69001.1	no	no
Studiervirinae	Caroctavirus	Citrobacter phage CR8	YP_009004183.1	no	no
Studiervirinae	Chatterjeevirus	Vibrio phage N4	YP_003347917.1	no	no
Studiervirinae	Chatterjeevirus	Vibrio phage ICP3	YP_004251265.1	no	no
Studiervirinae	Foetvirus	Escherichia phage SRT7	AXC34588.1	no	no
Studiervirinae	Ghunavirus	Pseudomonas phage shl2	CUR50692.1	no	no
Studiervirinae	Ghunavirus	Pseudomonas phage Henninger	AUV61734.1	no	no
Studiervirinae	Jarilovirus	Pectobacterium phage Jarilo	AWD92503.1	no	no
Studiervirinae	Kayfunavirus	Escherichia phage PE3-1	YP_009044272.1	no	no
Studiervirinae	Kayfunavirus	Cronobacter phage Dev2	YP_009005131.1	no	no
Studiervirinae	Kayfunavirus	Shigella phage SFPH2]	AXF40850.1	no	no
Studiervirinae	Minipunavirus	Morganella phage MmP1	YP_002048647.1	no	no
Studiervirinae	Ningirsuvirus	Dickeya phage Ninurta	AWD92642.1	no	no

subfamily	genus	species	GenBank accession no.	Presence/absence of 8-aa insertion	Phylogenetic affinity to PolIBCD+
Studiervirinae	Pektosvirus	Pectobacterium phage PP47	APW79766.1	no	no
Studiervirinae	Pektosvirus	Pectobacterium phage PP81	APU03048.1	no	no
Studiervirinae	Pektosvirus	Pectobacterium phage PPWS4	BBA26439.1	no	no
Studiervirinae	Pifdecavirus	Pseudomonas phage 22PfluR64PP	AWH14605.1	no	no
Studiervirinae	Pifdecavirus	Pseudomonas phage PFP1	AWY10472.1	no	no
Studiervirinae	Pijolavirus	Pseudomonas phage PspYZU08	ASD52203.1	no	no
Studiervirinae	Przondovirus	Enterobacter phage phiEap-1	YP_009196361.1	no	no
Studiervirinae	Przondovirus	Klebsiella virus KP32	AWN07108.1	no	no
Studiervirinae	Przondovirus	Klebsiella phage Pharr	QBZ71230.1	no	no
Studiervirinae	Teetrevirus	Citrobacter phage SH1	YP_009286652.1	no	no
Studiervirinae	Teetrevirus	Enterobacter phage E-2	YP_009226193.1	no	no
Studiervirinae	Teetrevirus	Leclercia phage 10164-302	ATA65265.1	no	no
Studiervirinae	Teseptimavirus	Yersinia phage vB_YenP_AP5	YP_009102815.1	no	no
Studiervirinae	Teseptimavirus	Pseudomonad phage gh-1	NP_813764.1	no	no
Studiervirinae	Teseptimavirus	Escherichia phage Ebrios	AVJ51906.1	no	no
	Bonnellvirus	Escherichia phage Lidtsur	QBZ71530.1	yes	yes
	Cuernavacavirus	Rhizobium phage RHEph02	AGC35595.1	yes	yes
	Cuernavacavirus	Rhizobium phage RHEph08	AGC35952.1	yes	yes
	Ermolevavirus	Escherichia phage PGT2	ATS92439.1	yes	yes
	Gajwadongvirus	Escherichia phage ECBP5	YP_009146393.1	no	no
	Gajwadongvirus	Pectobacterium phage PP99	APW79713.1	no	no
	Gyeongsanvirus	Ralstonia phage RsoP1EGY	AUO78210.1	no	no
	Jiaoyazivirus	Ralstonia phage RSB3	YP_008853911.1	yes	yes
	Kalppathivirus	Curvibacter phage P26059B	ASJ79303.1	yes	yes
	Kelmasvirus	Ralstonia phage RSB2	YP_009017751.1	no	no
	Lullwatervirus	Caulobacter phage Lullwater	ATI16325.1	yes	yes
	Maculvirus	Vibrio phage OWB	QIG66517.1	yes	yes
	Maculvirus	Vibrio phage VP93	YP_002875638.1	yes	yes
	Napahaivirus	Pseudomonas phage VSW-3	YP_009596163.1	yes	yes
	Paadamvirus	Rhizobium phage RHEph01	AGC35533.1	no	no
	Pelagivirus	Pelagibacter phage HTVC019P	YP_007517826.1	no	no
	Percyvirus	Caulobacter phage Percy	YP_009225254.1	yes	yes
	Pollyceevirus	Pseudomonas phage PollyC	YP_009622550.1	yes	yes
	Pradovirus	Xanthomonas phage f20-Xaj	YP_009275483.1	yes	yes
	Pradovirus	Xanthomonas phage XAJ24	AMW36116.1	yes	yes
	Pradovirus	Xylella phage Cota	CAB1282929.1	yes	yes
	Scottvirus	Sphingomonas phage Scott	AXN53756.1	yes	yes
	Stompelvirus	Ralstonia phage RPSC1	ATN92964.1	no	no
	Stopavirus	Pelagibacter phage HTVC011P	YP_007517777.1	no	no
	Tawavirus	Vibrio phage JSF7	APD18125.1	yes	yes
	Tiamatvirus	Prochlorococcus virus PSSP7	YP_006355438.1	no	no
	Tiilvirus	Synechococcus virus P60	NP_570330.1	no	no
	Voetvirus	Synechococcus virus Syn5	YP_001285436.1	no	no
	Wuhanvirus	Pasteurella phage PHB01	ASD51034.1	no	no
	Wuhanvirus	Pasteurella phage PHB02	ARV77590.1	no	no
