# Supplementary material for: A Draft Genome Assembly of Culex pipiens pallens (Diptera: Culicidae) Using PacBio Sequencing
Source: Genome Biol Evol. 2021 Jan 27;13(3):evab005. doi: 10.1093/gbe/evab005 (PMC7936019; doi:10.1093/gbe/evab005)
Supplement: evab005_Supplementary_Data [file evab005_supplementary_data.zip › Table_S2.pdf]

Harada Inagaki
Table S2.

Protein Name	Sequence MD5 digest	Seq	Analysis	Signature	Signature Description	Start location	Stop location	E-value	Status	Date		
unclassified_insertion_YP_009101367.1_DNA_polymerase_Enterobacteria_phage_J865_	48b282f6e87c486b4ebb5ca910a05f01	821	Pfam	PF00476	DNA polymerase family A	371	793	4.50E-32	T	12-06-2020	IPR001098	DNA-directed DNA polymerase, family A, palm domain
unclassified_insertion_QGH73766.1_DNAdirected_DNA_polymerase_Vibrio_phage_vB_VhaP_VH5_	7e5a358b861decdd7d748ff7d2aec2fe	782	Pfam	PF00476	DNA polymerase family A	373	744	1.10E-32	T	12-06-2020	IPR001098	DNA-directed DNA polymerase, family A, palm domain
Pradovirus_Xylella_phage_Cota_CAB1282929.1	483c7e3249278696a40af0ae93b07d00	784	Pfam	PF00476	DNA polymerase family A	363	746	3.40E-39	T	12-06-2020	IPR001098	DNA-directed DNA polymerase, family A, palm domain
unclassified_insertion_AUR95257.1_hypothetical_protein_NVP1204O_37_Vibrio_phage_1.204.O._10N.222.46.F12_	af042b6909c4d8dcf7b7a54de48a974f	786	Pfam	PF00476	DNA polymerase family A	364	770	7.50E-41	T	12-06-2020	IPR001098	DNA-directed DNA polymerase, family A, palm domain
unclassified_insertion_YP_007236331.2_DNA_polymerase_I_EC_2.7.7.7_phageassociated_Yersinia_phage_phi8018_	832ced33354153889d00e78968fe137f	817	Pfam	PF00476	DNA polymerase family A	391	778	1.30E-29	T	12-06-2020	IPR001098	DNA-directed DNA polymerase, family A, palm domain
Ermolevavirus_Escherichia_phage_PGT2_ATS92439.1	20ec817d2cc095ba84a7a5e455d58d95	797	Pfam	PF00476	DNA polymerase family A	369	746	2.40E-36	T	12-06-2020	IPR001098	DNA-directed DNA polymerase, family A, palm domain
unclassified_insertion_YP_009203173.1_DNA_polymerase_Yersinia_phage_vB_YenP_ISAO8_	6e85e3ff512ee086cb599fa887d799a9	814	Pfam	PF00476	DNA polymerase family A	380	778	2.80E-28	T	12-06-2020	IPR001098	DNA-directed DNA polymerase, family A, palm domain
unclassified_insertion_AQT27793.1_DNA_polymerase_I_Ralstonia_phage_RSPI1_	777bef24d5434a60028a6a90d0b012f5	808	Pfam	PF00476	DNA polymerase family A	386	760	1.00E-34	T	12-06-2020	IPR001098	DNA-directed DNA polymerase, family A, palm domain
Lullwatervirus_Caulobacter_phage_Lullwater_ATI16325.1	331332c354118cfa669b4bc7b06c2643	755	Pfam	PF00476	DNA polymerase family A	327	709	2.30E-36	T	12-06-2020	IPR001098	DNA-directed DNA polymerase, family A, palm domain
Pradovirus_Xanthomonas_phage_f20-Xaj_YP_009275483.1	03b6564271ab0450e7fef9dad71d92de	822	Pfam	PF00476	DNA polymerase family A	372	785	3.20E-39	T	12-06-2020	IPR001098	DNA-directed DNA polymerase, family A, palm domain
Melnykvirinae_Aerosvirus_Aeromonas_phage_ZPAH7_AZQ96410.1	3aa95b7b724592727c86743fe2d40c71	585	Pfam	PF00476	DNA polymerase family A	144	538	3.40E-30	T	12-06-2020	IPR001098	DNA-directed DNA polymerase, family A, palm domain
unclassified_insertion_ADD21653.1_DNA_polymerase_Caulobacter_phage_Cd1_	6756b55c98c94a9a401bf884fb10c782	797	Pfam	PF00476	DNA polymerase family A	366	754	1.30E-35	T	12-06-2020	IPR001098	DNA-directed DNA polymerase, family A, palm domain
unclassified_insertion_QIW87158.1_putative_DNA_polymerase_Vibrio_phage_vB_VpP_FE11_	a21f1f3c5c2370df7c85e864a9014074	808	Pfam	PF00476	DNA polymerase family A	446	800	4.80E-28	T	12-06-2020	IPR001098	DNA-directed DNA polymerase, family A, palm domain
unclassified_insertion_ASL24404.1_putative_DNA_polymerase_Alteromonas_virus_vB_AspPH4/4_	81cbe5168968c68a1b3fa6b60d10ca72	770	Pfam	PF00476	DNA polymerase family A	386	762	3.30E-33	T	12-06-2020	IPR001098	DNA-directed DNA polymerase, family A, palm domain
Okabevirinae_Mguuvirus_Burkholderia_phage_JG068_YP_008853857.1	8881f82a4da6c90b03f681f10d34f7fd	790	Pfam	PF00476	DNA polymerase family A	356	755	8.40E-35	T	12-06-2020	IPR001098	DNA-directed DNA polymerase, family A, palm domain
Percyvirus_Caulobacter_phage_Percy_YP_009225254.1	a252b2c7a32742539c02a507288dfc29	796	Pfam	PF00476	DNA polymerase family A	377	758	3.00E-34	T	12-06-2020	IPR001098	DNA-directed DNA polymerase, family A, palm domain
Slopekvirinae_Drulisvirus_Escherichia_phage_Minorna_QBP07090.1	886d4a745b318651d447ea7c45a001c3	783	Pfam	PF00476	DNA polymerase family A	340	775	3.80E-27	T	12-06-2020	IPR001098	DNA-directed DNA polymerase, family A, palm domain
Okabevirinae_Risjevirus_Ralstonia_phage_RSJ2_YP_009216556.1	b7f527b4d0ccbfa26e19fdaa6e0986b0	791	Pfam	PF00476	DNA polymerase family A	358	750	2.10E-31	T	12-06-2020	IPR001098	DNA-directed DNA polymerase, family A, palm domain
Corkvirinae_Phimunavirus_Pectobacterium_phage_Peat1_YP_009224652.1	0254f458539cb1b70de0acacd00ddc34	780	Pfam	PF00476	DNA polymerase family A	386	770	1.70E-30	T	12-06-2020	IPR001098	DNA-directed DNA polymerase, family A, palm domain
Tawavirus_Vibrio_phage_JSF7_APD18125.1	543bbd8a5c06323f6a9a2c438bab5e8b	797	Pfam	PF00476	DNA polymerase family A	405	788	6.90E-30	T	12-06-2020	IPR001098	DNA-directed DNA polymerase, family A, palm domain
Jiaoyazivirus_Ralstonia_phage_RSB3_YP_008853911.1	393ea5460289140d42bd4baa0b5ede3e	836	Pfam	PF00476	DNA polymerase family A	452	817	6.60E-27	T	12-06-2020	IPR001098	DNA-directed DNA polymerase, family A, palm domain
Kalppathivirus_Curvibacter_phage_P26059B_ASJ79303.1	d2105c0b5977f7b4986ab6c061ec628f	811	Pfam	PF00476	DNA polymerase family A	375	774	8.30E-29	T	12-06-2020	IPR001098	DNA-directed DNA polymerase, family A, palm domain
unclassified_insertion_AGC35656.1_putative_DNAdirected_DNA_polymerase_protein_Rhizobium_phage_RHEph03_	6a3473559875a3b6e4dad7cde8317f60	798	Pfam	PF00476	DNA polymerase family A	383	781	1.90E-36	T	12-06-2020	IPR001098	DNA-directed DNA polymerase, family A, palm domain
Cuernavacavirus_Rhizobium_phage_RHEph02_AGC35595.1	6a3473559875a3b6e4dad7cde8317f60	798	Pfam	PF00476	DNA polymerase family A	383	781	1.90E-36	T	12-06-2020	IPR001098	DNA-directed DNA polymerase, family A, palm domain
unclassified_insertion_AFE86116.1_DNA_polymerase_Enterobacter_phage_phiKDA1_	27e84b778859bfbbe1a4d3aa13bceaec	794	Pfam	PF00476	DNA polymerase family A	342	777	3.60E-28	T	12-06-2020	IPR001098	DNA-directed DNA polymerase, family A, palm domain
Pradovirus_Xanthomonas_phage_XAJ24_AMW36116.1	d208fb7041800df96ae12c66e2a8a6b8	825	Pfam	PF00476	DNA polymerase family A	375	778	1.30E-33	T	12-06-2020	IPR001098	DNA-directed DNA polymerase, family A, palm domain
Bonnellvirus_Escherichia_phage_Lidtsur_QBZ71530.1	491308eda03f00f99e0af251c0349f5d	819	Pfam	PF00476	DNA polymerase family A	367	791	2.20E-30	T	12-06-2020	IPR001098	DNA-directed DNA polymerase, family A, palm domain
unclassified_insertion_YP_009223394.1_DNA_polymerase_I_Cronobacter_phage_DevCD23823_	78ed41dcc4b18746e982f0ac3e2e815a	821	Pfam	PF00476	DNA polymerase family A	437	785	6.70E-31	T	12-06-2020	IPR001098	DNA-directed DNA polymerase, family A, palm domain
unclassified_insertion_QHJ75317.1_DNA_polymerase_Sphaerotilus_phage_vB_SnaPR1_	343e6114a7cdf8a0a19d68d0829f84b9	811	Pfam	PF00476	DNA polymerase family A	364	804	9.10E-31	T	12-06-2020	IPR001098	DNA-directed DNA polymerase, family A, palm domain
Melnykvirinae_Ahphunavirus_Aeromonas_phage_CF7_ASZ71994.1	efbda5e36cda34229783be43c3f81453	828	Pfam	PF00476	DNA polymerase family A	372	780	6.80E-31	T	12-06-2020	IPR001098	DNA-directed DNA polymerase, family A, palm domain
Corkvirinae_Phimunavirus_Pectobacterium_phage_PP90_YP_009289625.1	75ecc88d25787e809bb0e4d6dd41c0b2	795	Pfam	PF00476	DNA polymerase family A	388	772	1.90E-31	T	12-06-2020	IPR001098	DNA-directed DNA polymerase, family A, palm domain
unclassified_insertion_YP_009103233.1_putative_DNA_polymerase_Acinetobacter_phage_vB_AbaP_Acibel007_	aef00f7a3a21a0a9e56805bc826f26db	768	Pfam	PF00476	DNA polymerase family A	351	731	9.50E-31	T	12-06-2020	IPR001098	DNA-directed DNA polymerase, family A, palm domain
unclassified_insertion_QDH85472.1_DNA_polymerase_Proteus_phage_vB_PmiP_RS8pmA_	c646e8b3d394e3a9181638ab94ce047e	804	Pfam	PF00476	DNA polymerase family A	434	782	1.90E-27	T	12-06-2020	IPR001098	DNA-directed DNA polymerase, family A, palm domain
Beijerinckvirinae_Friunavirus_Acinetobacter_phage_AB3_YP_008060158.1	2f8a6f9cbd4266c9a9a6d8bf17b9e63c	777	Pfam	PF00476	DNA polymerase family A	360	741	3.80E-32	T	12-06-2020	IPR001098	DNA-directed DNA polymerase, family A, palm domain
Napahaivirus_Pseudomonas_phage_VSW-3_YP_009596163.1	f9397776d80aa8d09f60216b1f76d0c5	775	Pfam	PF00476	DNA polymerase family A	351	744	4.50E-34	T	12-06-2020	IPR001098	DNA-directed DNA polymerase, family A, palm domain
unclassified_insertion_ASV44561.1_DNA_polymerase_I_Agrobacterium_phage_Atu_ph02_	08419fa7852ec5aeeeadb281b4ee0e00	786	Pfam	PF00476	DNA polymerase family A	358	755	3.40E-35	T	12-06-2020	IPR001098	DNA-directed DNA polymerase, family A, palm domain
unclassified_insertion_QDH84547.1_putative_DNA_polymerase_I_Achromobacter_phage_vB_AxyP_1932_Axy21_	63233646f5166e363607dc5501b12e2c	786	Pfam	PF00476	DNA polymerase family A	441	778	5.60E-34	T	12-06-2020	IPR001098	DNA-directed DNA polymerase, family A, palm domain
unclassified_insertion_YP_007348339.1_DNA_polymerase_Cronobacter_phage_vB_CskP_GAP227_	657d6f9efecc586b7c29ae10f8e52267	822	Pfam	PF00476	DNA polymerase family A	379	787	1.00E-31	T	12-06-2020	IPR001098	DNA-directed DNA polymerase, family A, palm domain
Maculvirus_Vibrio_phage_VP93_YP_002875638.1	ffd99cf1d6a250a9480309694cd3ba8c	811	Pfam	PF00476	DNA polymerase family A	450	803	4.90E-28	T	12-06-2020	IPR001098	DNA-directed DNA polymerase, family A, palm domain
Krylovirinae_Phikmvvirus_Pseudomonas_phage_phiKMV_NP_877458.1	64fadc598f14d78128d49e864a6151af	807	Pfam	PF00476	DNA polymerase family A	398	799	3.30E-30	T	12-06-2020	IPR001098	DNA-directed DNA polymerase, family A, palm domain
unclassified_insertion_QIW89945.1_DNA_polymerase_Aeromonas_phage_PS_	38b00ff993e86eb370486ac97fe735d5	774	Pfam	PF00476	DNA polymerase family A	359	736	8.70E-29	T	12-06-2020	IPR001098	DNA-directed DNA polymerase, family A, palm domain
unclassified_insertion_AVI05039.1_DNA_polymerase_I_Salmonella_phage_vB_SpuP_Spp16_	5870614c213369be83ce3497f4f88df4	824	Pfam	PF00476	DNA polymerase family A	380	786	1.80E-32	T	12-06-2020	IPR001098	DNA-directed DNA polymerase, family A, palm domain
unclassified_insertion_QEG09739.1_DNA_polymerase_Stenotrophomonas_phage_Ponderosa_	d0da4ab99fb9b12e27d5606d8404bcbf	783	Pfam	PF00476	DNA polymerase family A	359	746	4.00E-43	T	12-06-2020	IPR001098	DNA-directed DNA polymerase, family A, palm domain
Krylovirinae_Phikmvvirus_Pantoea_phage_LIMEzero_YP_004539096.1	0278f5d762fd9dcddf3b566c0af42780	814	Pfam	PF00476	DNA polymerase family A	401	784	8.50E-28	T	12-06-2020	IPR001098	DNA-directed DNA polymerase, family A, palm domain
Okabevirinae_Higashivirus_Ralstonia_phage_RsoP1IDN_AUG85414.1	6e8717505fb77d57684d186b01efe211	734	Pfam	PF00476	DNA polymerase family A	307	689	7.80E-30	T	12-06-2020	IPR001098	DNA-directed DNA polymerase, family A, palm domain
Slopekvirinae_Drulisvirus_Klebsiella_phage_KpV71_YP_009302723.1	2e2ff05c884746616d3f178449da640e	803	Pfam	PF00476	DNA polymerase family A	352	794	8.50E-29	T	12-06-2020	IPR001098	DNA-directed DNA polymerase, family A, palm domain
Melnykvirinae_Aerosvirus_Aeromonas_phage_25AhydR2PP_AWH15425.1	f2990b1ba3d53eafa9c2e3d6a8eb01cd	828	Pfam	PF00476	DNA polymerase family A	387	781	9.90E-30	T	12-06-2020	IPR001098	DNA-directed DNA polymerase, family A, palm domain
unclassified_insertion_YP_008858892.1_DNA_polymerase_A_Xylella_phage_Paz_	b56bb8a1cb68a253b1d1d6892f87c58c	786	Pfam	PF00476	DNA polymerase family A	365	749	2.70E-40	T	12-06-2020	IPR001098	DNA-directed DNA polymerase, family A, palm domain
Slopekvirinae_Drulisvirus_Klebsiella_phage_KPV811_APD20688.1	0d0de43419693f390a73aa73da3ca047	788	Pfam	PF00476	DNA polymerase family A	342	780	1.80E-27	T	12-06-2020	IPR001098	DNA-directed DNA polymerase, family A, palm domain
Beijerinckvirinae_Friunavirus_Acinetobacter_phage_phiAB1_YP_009189359.1	3e99e1d04c3ccf9abbe690f95ab16f99	494	Pfam	PF00476	DNA polymerase family A	77	458	1.20E-33	T	12-06-2020	IPR001098	DNA-directed DNA polymerase, family A, palm domain
Melnykvirinae_Ahphunavirus_Aeromonas_phage_Ahp1_ALP47739.1	1cc7ca95ef05a0c83cbad4b102df9fe7	828	Pfam	PF00476	DNA polymerase family A	372	780	5.30E-30	T	12-06-2020	IPR001098	DNA-directed DNA polymerase, family A, palm domain
Melnykvirinae_Wanjuvirus_Pectobacterium_phage_PP2_AOT25385.1	848d46a632867c83119c53602751d1fe	818	Pfam	PF00476	DNA polymerase family A	366	781	1.40E-32	T	12-06-2020	IPR001098	DNA-directed DNA polymerase, family A, palm domain
unclassified_insertion_QDH46280.1_putative_polymerase_Aeromonas_phage_LAh2_	915f3f467a916544ac1d015db3378e4e	830	Pfam	PF00476	DNA polymerase family A	372	782	2.20E-30	T	12-06-2020	IPR001098	DNA-directed DNA polymerase, family A, palm domain
unclassified_insertion_QAX92385.1_DNA_polymerase_Providencia_phage_vB_PstP_Stuart_	370dcd4a977bead29eb3eb75a74d5cb3	790	Pfam	PF00476	DNA polymerase family A	387	782	9.00E-24	T	12-06-2020	IPR001098	DNA-directed DNA polymerase, family A, palm domain
unclassified_insertion_QHR72957.1_DNA_polymerase_I_Escherichia_phage_usur_	81990a1106793b0e7c927b83f5e9d8e3	821	Pfam	PF00476	DNA polymerase family A	371	793	7.50E-31	T	12-06-2020	IPR001098	DNA-directed DNA polymerase, family A, palm domain
Corkvirinae_Phimunavirus_Pectobacterium_phage_vB_PatP_CB5_YP_009625539.1	259324232c2f5c96f86547ce0db0e8b2	780	Pfam	PF00476	DNA polymerase family A	425	773	3.80E-32	T	12-06-2020	IPR001098	DNA-directed DNA polymerase, family A, palm domain
Pollyceevirus_Pseudomonas_phage_PollyC_YP_009622550.1	b6f0c3ac84f62e185c79e36183a9cbb1	772	Pfam	PF00476	DNA polymerase family A	352	732	7.60E-37	T	12-06-2020	IPR001098	DNA-directed DNA polymerase, family A, palm domain
unclassified_insertion_CCI88403.2_DNA_polymerase_I_phageassociated_Yersinia_phage_phiR801_	701fa341f2eafcd051772720aa97c446	815	Pfam	PF00476	DNA polymerase family A	372	779	2.90E-29	T	12-06-2020	IPR001098	DNA-directed DNA polymerase, family A, palm domain
unclassified_insertion_QGF20958.1_DNA_polymerase_I_Pectobacterium_phage_MA13_	94fa45568f9c0273d51061e52cb43380	822	Pfam	PF00476	DNA polymerase family A	439	783	1.60E-29	T	12-06-2020	IPR001098	DNA-directed DNA polymerase, family A, palm domain
unclassified_insertion_APU00317.1_DNA_polymerase_I_Ralstonia_phage_RSPII1_	ed773f58039538d91e6c776cba094b26	816	Pfam	PF00476	DNA polymerase family A	399	771	8.70E-31	T	12-06-2020	IPR001098	DNA-directed DNA polymerase, family A, palm domain
unclassified_insertion_YP_007007774.1_putative_DNA_polymerase_Aeromonas_phage_phiAS7_	668aaecaf679e8588f258f3c1e1dcbdb	828	Pfam	PF00476	DNA polymerase family A	387	781	3.50E-30	T	12-06-2020	IPR001098	DNA-directed DNA polymerase, family A, palm domain
Cuernavacavirus_Rhizobium_phage_RHEph08_AGC35952.1	9e0426c1bfbd5ebfa52fc68de3266d6e	798	Pfam	PF00476	DNA polymerase family A	383	781	9.80E-37	T	12-06-2020	IPR001098	DNA-directed DNA polymerase, family A, palm domain
unclassified_insertion_YP_009275048.1_putative_DNA_polymerase_Pseudomonas_phage_YMC11/06/C171_PPU_BP_	139aa2c4a8f3ad0f1d47709bb2a1e50a	784	Pfam	PF00476	DNA polymerase family A	379	777	5.60E-34	T	12-06-2020	IPR001098	DNA-directed DNA polymerase, family A, palm domain
Scottvirus_Sphingomonas_phage_Scott_AXN53756.1	e554fa2cedbe48fb6c46396163438bfe	830	Pfam	PF00476	DNA polymerase family A	393	782	1.60E-35	T	12-06-2020	IPR001098	DNA-directed DNA polymerase, family A, palm domain
unclassified_insertion_APU03166.1_DNA_polymerase_Ralstonia_virus_phiAp1_	e33c5b6d7d7ed711463ca71ccf2e6b68	830	Pfam	PF00476	DNA polymerase family A	447	819	1.60E-26	T	12-06-2020	IPR001098	DNA-directed DNA polymerase, family A, palm domain
Beijerinckvirinae_Friunavirus_Acinetobacter_phage_Fri1_YP_009203032.1	ffd5dd8a77872bd4dac84291abe8e1b3	486	Pfam	PF00476	DNA polymerase family A	69	450	7.90E-33	T	12-06-2020	IPR001098	DNA-directed DNA polymerase, family A, palm domain
Krylovirinae_Phikmvvirus_Pseudomonas_phage_LKA1_YP_001522870.1	dfda1013758be1b5c8761dc4d6b583f9	791	Pfam	PF00476	DNA polymerase family A	388	783	8.80E-36	T	12-06-2020	IPR001098	DNA-directed DNA polymerase, family A, palm domain
Maculvirus_Vibrio_phage_OWB_QIG66517.1	1eafa77c66a8a1102217c7f65073a8ac	808	Pfam	PF00476	DNA polymerase family A	451	800	2.90E-28	T	12-06-2020	IPR001098	DNA-directed DNA polymerase, family A, palm domain
Okabevirinae_Risjevirus_Ralstonia_phage_RSJ5_YP_009218109.1	82a7234a489964a03c17186da604b76f	791	Pfam	PF00476	DNA polymerase family A	358	750	2.30E-31	T	12-06-2020	IPR001098	DNA-directed DNA polymerase, family A, palm domain
unclassified_insertion_ANS06219.1_hypothetical_protein_Phage_MedPESWcelC56_	e0f35cfd89ad4a177f61e8d38946ef49	783	Pfam	PF00476	DNA polymerase family A	401	774	5.50E-34	T	12-06-2020	IPR001098	DNA-directed DNA polymerase, family A, palm domain
unclassified_insertion_YP_007006588.1_DNA_polymerase_I_Escherichia_phage_phiKT_	f9148562457df8711810d9a6ae79e58b	797	Pfam	PF00476	DNA polymerase family A	369	753	3.90E-37	T	12-06-2020	IPR001098	DNA-directed DNA polymerase, family A, palm domain
Melnykvirinae_Wanjuvirus_Pectobacterium_phage_Arno160_AZF88083.1	888f2a8665c1685ed9a5b01e8bc5f134	818	Pfam	PF00476	DNA polymerase family A	366	781	1.60E-32	T	12-06-2020	IPR001098	DNA-directed DNA polymerase, family A, palm domain
Okabevirinae_Higashivirus_Ralstonia_phage_RSB1_YP_002213707.1	b239b7bbd316ab50758e2c592db6e9cb	815	Pfam	PF00476	DNA polymerase family A	390	772	1.40E-28	T	12-06-2020	IPR001098	DNA-directed DNA polymerase, family A, palm domain
unclassified_insertion_AVH86128.1_putative_DNA_polymerase_I_Pseudomonas_phage_phiNV3_	28f80691e0f0c19b67d479b77f76682b	786	Pfam	PF00476	DNA polymerase family A	422	776	5.40E-26	T	12-06-2020	IPR001098	DNA-directed DNA polymerase, family A, palm domain
